# Trajectories of Individuating and Relating Autonomy in Adolescence: A Longitudinal Study in a Relational Cultural Context

**DOI:** 10.1007/s10964-025-02298-2

**Published:** 2025-12-05

**Authors:** Chao-Sheng Kuo, Yih-Lan Liu

**Affiliations:** Institute of Education, National Yang Ming Chiao Tung University (NYCU), 1001 University Road, Hsinchu, Taiwan R.O.C.

**Keywords:** Dual autonomy, Adolescent development, Relational culture, Gender differences, Latent growth modeling, Bayesian analysis

## Abstract

Autonomy is increasingly understood as a relationally embedded process rather than a pursuit of independence, yet few longitudinal studies track how volition develops across cultural and developmental contexts. This study examined the dual trajectories of individuating autonomy and relating autonomy across adolescence and educational transitions. Participants were 777 Taiwanese adolescents (48.8% female; *M* = 15.22 years, *SD* = 1.56), including junior high (*n* = 371) and senior high (*n* = 406) cohorts, each followed across four waves to yield eight cohort-sequential waves. Latent growth and Bayesian autoregressive models were used to estimate developmental change and subgroup differences by gender and educational stage. Individuating autonomy was stable across adolescence, whereas relating autonomy followed a curvilinear course—declining and later recovering—most evident among girls and senior high students. Boys and junior high students reported higher initial levels on both autonomy forms. The combined cohort model indicated gradual declines and synchronous short-term fluctuations, highlighting intraindividual variability beyond linear trends. Findings suggest that autonomy in relationally interdependent societies develops through differentiated yet interconnected pathways, reflecting dynamic coordination between self-endorsed action and relational attunement. Results deepen the application of Self-Determination Theory by demonstrating that volition is both culturally grounded and universally relevant—enacted within, rather than apart from, enduring relationships.

## Introduction

Adolescence is a critical period for developing autonomy, yet how volition unfolds as youth navigate social expectations, educational transitions, and relational obligations remains poorly understood. Most developmental research has conceptualized autonomy as a general construct, overlooking how its distinct forms may change dynamically across time and context. In particular, little is known about how adolescents balance self-endorsed agency with relational harmony during school transitions, when increasing parental expectations and academic demands intensify the tension between individuality and connectedness. This study examines the longitudinal development of two interrelated forms of autonomy—individuating and relating autonomy—among Taiwanese adolescents, clarifying how volitional functioning develops within relationally interdependent cultural contexts.

Building on Self-Determination Theory (SDT), autonomy is conceptualized as volitional functioning—acting with psychological freedom and personal endorsement within meaningful relationships (Ryan & Deci, 2017). Rather than standing in opposition to relatedness, autonomy emerges through socialization processes that facilitate internalization and perspective taking (Deci & Ryan, 2000). When parents and teachers provide rationales, acknowledge adolescents’ viewpoints, and encourage shared decision-making, youth are more likely to act volitionally while maintaining relational harmony (Reeve, 2016; Soenens & Vansteenkiste, 2020). Empirical evidence from East Asian settings further demonstrates that autonomy-supportive environments promote adaptive functioning and well-being, indicating that benefits of autonomy extend beyond individualistic cultural contexts (Bao & Lam, 2008; Chirkov, 2011). However, most SDT-based studies in these contexts have relied on global assessments of autonomy, typically treating volitional functioning as a unitary construct across personal and relational domains.

The dual autonomy framework (Yeh & Yang, 2006) distinguishes between two interrelated forms of volitional functioning: individuating autonomy—the pursuit of self-endorsed goals and personal agency—and relating autonomy—acting volitionally while maintaining close interpersonal ties. Both reflect volitional functioning but differ in orientation and developmental implications. Individuating autonomy aligns with self-regulation and self-efficacy, whereas relating autonomy supports empathy and relational adjustment (Wu & Yeh, 2015; Yeh et al., 2016). This framework extends SDT by specifying how autonomy operates as both an individual and relational capacity within interdependent cultural contexts. It also aligns with foundational Western theories that view autonomy as relationally grounded rather than opposed to connectedness (Grotevant & Cooper, 1986; Hauser et al., 1984; Kagitcibasi, 2005; Ryan, 1993), offering a theoretical bridge between culturally embedded and universal perspectives on volition. Although empirical support for the dual autonomy framework has accumulated, much of this work has examined individuating and relating autonomy using cross-sectional designs that capture autonomy at single time points (Wu & Yeh, 2015; Zimmer-Gembeck & Collins, 2006).

Individuating and relating autonomy can also be understood through cognitive, functional, and emotional components (Noom et al., 2001). Cognitively, youth evaluate the potential consequences of their choices; functionally, they regulate and implement these decisions; emotionally, they experience confidence and congruence with their actions. This tripartite structure provides a developmentally grounded and culturally sensitive framework for assessing volitional functioning. It further links the dual autonomy framework with broader developmental theories, by illustrating how autonomy evolves as adolescents integrate reasoning, self-regulation, and emotion across time and within relational contexts.

Autonomy also develops within specific domains and relationships. Social-Cognitive Domain Theory (Smetana, 2017) posits that adolescents assert autonomy in personal domains (e.g., friendships, clothing) while accepting parental authority in moral or conventional domains. Similarly, the Governance Transfer Model (Tilton-Weaver & Marshall, 2017) conceptualizes autonomy development as a co-regulatory process in which parents gradually transfer decision-making authority as youth demonstrate competence—particularly in low-stakes areas such as leisure or food choices, while maintaining oversight in high-stakes domains like academics or health. Collectively, these frameworks converge on a developmental view of autonomy as a process that is negotiated rather than granted, emphasizing adolescents’ active role in redefining the boundaries of decision-making within relationships. These perspectives align with the dual autonomy framework: individuating autonomy centers on self-direction, whereas relating autonomy emphasizes volitional harmony within relationships. Yet, most empirical studies have examined these dynamics using cross-sectional designs (Zimmer-Gembeck & Collins, 2006), focusing on autonomy at specific moments rather than across developmental periods. A longitudinal approach is therefore essential to capture the developmental processes through which autonomy becomes embedded within relational contexts.

Evidence from Taiwan supports this dual-process perspective. Individuating autonomy relates to self-regulation and internal control, whereas relating autonomy predicts empathy and relational adjustment (Wu & Yeh, 2015). Longitudinal findings indicate that autonomy-supportive parenting promotes adaptive functioning through the growth of both forms of autonomy, which in turn mediate well-being over time (Yeh et al., 2016). Such evidence not only validates the dual autonomy framework within a Confucian-heritage context but also refines developmental theory by illustrating how volitional functioning remains adaptive within relational obligations. These findings highlight that autonomy operates as a culturally situated yet universal developmental process, consistent with SDT’s view of volition as enacted through connectedness rather than separation.

Despite these advances, prior research has primarily described individuating and relating autonomy at specific developmental points—particularly surrounding school transitions—without tracing how these forms shift in relation to changing relational expectations and identity demands. In the broader autonomy literature, many studies have relied on cross-sectional or domain-general approaches (Soenens et al., 2017; Yeh & Yang, 2006), characterizing volitional functioning at specific moments rather than across extended developmental periods. Longitudinal data are therefore essential for understanding how individuating and relating autonomy co-develop across adolescence within culturally embedded relationships.

Autonomy development may unfold differently across subgroups due to gendered socialization and shifting developmental contexts. In Confucian-heritage contexts, girls are often encouraged to value relational harmony, whereas boys are socialized toward self-direction and achievement (Cheng, 2024; Way et al., 2014). Developmental contexts also vary across school stage: junior high students typically experience greater parental control, while senior high students encounter heightened academic pressure and identity challenges (Noom et al., 2001; Shah et al., 2023). Comparing these subgroups can clarify whether variations in volitional functioning reflect gendered role expectations or normative developmental adaptation. Examining these dynamics within a relationally interdependent culture also provides an empirically meaningful context for evaluating the universality of autonomy-as-volition proposed by SDT, particularly in understanding how autonomy develops within relationally embedded relationships.

## Current Study

A central unresolved issue in the literature is how the two theoretically distinct forms of volitional functioning identified in Self-Determination Theory and the dual autonomy framework—individuating and relating autonomy—unfold across adolescence within relationally interdependent cultural contexts. In SDT, autonomy is conceptualized as relationally embedded volition rather than independence, and the dual autonomy framework further specifies this volitional process into self-directed (individuating autonomy) and relationship-oriented (relating autonomy) forms; however, how these forms develop over the course of adolescence remains unclear. Building on the theoretical and empirical foundations outlined above, this study was grounded in SDT and the dual autonomy framework to examine these developmental processes. The current study investigated whether individuating and relating autonomy develop in synchrony or along distinct trajectories across adolescence (Research Question 1). It also explored whether gender and educational stage moderate these developmental trajectories, reflecting contextual differences in socialization and schooling experiences (Research Question 2). Finally, the study investigated how autonomy is constructed and differentiated within interdependent relationships that characterize Confucian-heritage cultures (Research Question 3). Together, these aims address the need to conceptualize autonomy development as a culturally situated yet universal process that integrates personal agency with relational connectedness.

## Methods

### Participants

The final analytic sample consisted of 777 Taiwanese adolescents aged between 12.9 and 18.1 years (*M* age = 15.22, *SD* = 1.56), recruited from 10 junior and senior high schools across northern, central, and southern Taiwan. Participants were drawn from a larger cohort of 863 adolescents enrolled in a national longitudinal project funded by the National Science Council. Adolescents who provided only one wave of data or lacked basic demographic information were excluded from the final sample.

The analytic sample included 371 junior high school students (216 boys [58.2%], 155 girls [41.8%]; *M* age = 13.68, *SD* = 0.30) and 406 senior high/vocational school students (182 boys [44.8%], 224 girls [55.2%]; *M* age = 16.67, *SD* = 0.39), representing 47.7% and 52.3% of the total sample (*N* = 777), respectively. Across the entire sample, boys (*n* = 398, 51.2%) had a mean age of 15.07 years (*SD* = 1.54), while girls (*n* = 379, 48.8%) had a mean age of 15.39 years (*SD* = 1.58). Junior high participants were followed from 7th to 8th grade, and senior high participants from 10th to 11th grade. Data collection occurred once per semester over two academic years (2010–2011), resulting in four waves of data for each cohort. The resulting cohort-sequential design allowed the construction of an eight-wave dataset capturing developmental change from early to late adolescence.

Most adolescents (74.26%) reported living with both parents, with others living only with their mother (11.71%), only with their father (9.65%), or with other caregivers such as grandparents or relatives (4.38%). Regarding parental marital status, 76.06% of adolescents reported that their parents were married and cohabiting, 14.67% were divorced, and the remaining were separated, widowed, or unmarried but cohabiting. In terms of parental education, approximately 45% of fathers and 54% of mothers completed senior high or vocational education, and around 22% of fathers and 18% of mothers had post-secondary degrees. A summary of all demographic variables is presented in Table 1.

**Table 1:** Basic Demographic Information of Participants

Background Information and Levels	N	Percentage (%)
**Gender**		
Male	398	51.22
Female	379	48.78
**Educational Stage**		
Junior High School	371	47.76
Senior High/Vocational School	406	52.25
**Living Situation with Parents**		
Living with Both Parents	577	74.26
Living with Father Only	75	9.65
Living with Mother Only	91	11.71
Other	34	4.38
**Parental Marital Status**		
Married and Living Together	591	76.06
Married and Separated	31	3.99
Unmarried and Living Together	6	0.77
Divorced	114	14.67
Widowed	34	4.38
Missing Data	1	0.13
**Father’s Educational Level**		
Elementary School or Below	43	5.53
Junior High School	185	23.81
Senior High/Vocational School	350	45.05
College, University, or Above	168	21.62
Other	14	1.80
Missing Data	17	2.19
**Mother’s Educational Level**		
Elementary School or Below	55	7.08
Junior High School	142	18.28
Senior High/Vocational School	418	53.80
College, University, or Above	138	17.76
Other	13	1.67
Missing Data	11	1.42

### Measures

#### Dual Autonomy

Adolescents’ dual autonomy was assessed using the Short Form of the Adolescent Autonomy Scale (Yeh, 2014), which includes two six-item subscales measuring individuating autonomy (IA) and relating autonomy (RA). Each subscale contains two items from each of the three components—cognitive, functional, and emotional—reflecting volitional functioning across different domains. For IA, example items include “I always know what I really want” (cognitive) and “Trying things I have never done before is not difficult for me” (functional). For RA, example items include “Even when I have my own ideas, I listen to my parents’ opinions when making important decisions” (cognitive) and “I can calmly discuss issues in my relationship with my parents” (emotional).

All items were rated on a 6-point Likert scale (1 = strongly disagree to 6 = strongly agree), with higher scores indicating greater autonomy. Internal consistency across the four waves was satisfactory to high (Cronbach’s α = 0.84–0.88 for IA; α = 0.86–0.90 for RA). The 12-item short form was adapted from the original 36-item Adolescent Autonomy Scale (Yeh & Yang, 2006) to reduce participant burden in longitudinal administration. Item selection for the short form was based on prior item analysis and confirmatory factor validation by (Yeh, 2014), which retained the six items with the highest factor loadings and conceptual representativeness from each subscale. Previous research has supported its factorial validity (Wu & Yeh, 2015). The measurement model demonstrated scalar invariance across the four waves, supporting its appropriateness for longitudinal comparisons. Demographic variables (gender, age, parental education, family structure) were also collected at baseline.

### Analysis Strategies

This study adopted a two-tiered longitudinal approach to examine the development of adolescents’ dual autonomy over time. First, latent growth modeling (LGM) was employed to examine the intra-individual trajectories of individuating and relating autonomy across four time points and to compare developmental patterns across gender and educational stage via multi-group models. LGM is particularly suitable for capturing both linear and nonlinear trends in psychological development while accounting for inter-individual variability (Curran et al., 2010). Subsequently, to take advantage of the extended developmental window afforded by the cohort-sequential design and to address structural missingness across waves, a Bayesian autoregressive latent growth model (ALGM) was implemented. ALGM enables the modeling of complex longitudinal dependencies and is well-suited to situations with partially overlapping cohorts and unbalanced data structures (McNeish, 2016; Van de Schoot et al., 2021). This combination of LGM and ALGM allowed for rigorous analysis of short-term growth patterns and the approximation of long-term developmental trends in autonomy across early to late adolescence.

This longitudinal study utilized self-report data collected across four waves from junior and senior high school students over a two-year period. Adolescents responded to survey instruments assessing their autonomy-related experiences at each wave. To evaluate the factorial validity of the measurement model, confirmatory factor analyses (CFAs) were conducted separately for each wave. Longitudinal measurement invariance was then tested across time, gender, and educational stage to ensure comparability of constructs across repeated measurements and demographic groups. Missing data were addressed using Full Information Maximum Likelihood (FIML) under the missing at random (MAR) assumption (Muthén & Muthén, 2012), allowing for valid estimation with partially observed longitudinal data.

After establishing scalar invariance, a bivariate latent growth model (LGM) was specified to examine the developmental trajectories of IA and RA. The model was designed to simultaneously estimate latent intercepts, linear slopes, and quadratic terms, along with their covariances. Model fit indices and likelihood-ratio comparisons were subsequently used to evaluate whether IA and RA were better characterized by linear or nonlinear trends. To explore subgroup differences, multi-group LGMs were conducted for gender and educational stage. These models assessed potential variation in the initial levels and growth parameters of IA and RA across groups, offering a basis for identifying developmental heterogeneity.

To extend the developmental window beyond the two-year observation period and to capture broader trends from early to late adolescence, we implemented a Bayesian autoregressive latent growth model (ALGM) using a cohort-sequential design. Specifically, junior high school students were recruited in 7th grade and followed through 8th grade (Waves 1–4), while senior high school students were recruited in 10th grade and followed through 11th grade (Waves 5–8). Although these cohorts were independent, their measurement waves were temporally aligned and conceptually continuous. This structure permitted the modeling of eight nominal time points, enabling a quasi-longitudinal analysis of adolescent autonomy development across a broader age span.

Preliminary analyses confirmed that the terminal scores of junior high students closely approximated the initial scores of senior high students, supporting the assumption of cohort comparability. A planned missingness design was employed to construct a pseudo-panel in which each participant contributed data for four consecutive waves. Within this framework, the ALGM incorporated autoregressive paths to model temporal dependencies across adjacent waves, thereby capturing cohort-overlapping continuity even in the absence of within-individual observations across all time points (McNeish, 2016).

To address the lack of data from Grade 9, we specified non-equidistant time scores—0, 0.5, 1, 1.5, 3, 3.5, 4, and 4.5—skipping over the unobserved 2-year period between 8th and 10th grades. This approach is consistent with established practices in planned missing data designs and autoregressive growth modeling (Curran et al., 2010; Enders, 2022; McNeish, 2016), and allows for a theoretically coherent approximation of developmental continuity across cohorts.

To better accommodate the complex data structure and capture nuanced developmental patterns, Bayesian estimation was adopted for its robustness in handling partially observed data (Van de Schoot et al., 2021). It also provides more stable parameter estimation and posterior inference under complex modeling conditions, including those involving heterogeneity in group composition (Zitzmann et al., 2022). Due to the structural confounding between educational stage and wave assignment, only gender was included as a time-invariant covariate in the ALGM. All models were estimated in Mplus 7.0, using maximum likelihood estimation for the LGM and multi-group models, and Bayesian estimation for the ALGM.

## Results

### Descriptive Statistics and Correlations

Table 2 presents the descriptive statistics and intercorrelations for individuating autonomy (IA) and relating autonomy (RA) across four measurement waves (T1–T4). All variables demonstrated acceptable levels of skewness and kurtosis, supporting the assumption of normality. Within each wave, IA and RA were moderately to strongly correlated (*r*s ranging from 0.548–0.635, all *p*s < 0.01), indicating synchronous fluctuations despite conceptual distinctiveness.

**Table 2:** Correlations and descriptive statistics of all variables

NO.	Variables	1	2	3	4	5	6	7	8
1	T1 IA	1							
2	T1 RA	0.586**	1						
3	T2 IA	0.491**	0.325**	1					
4	T2 RA	0.315**	0.533**	0.548**	1				
5	T3 IA	0.443**	0.329**	0.521**	0.310**	1			
6	T3 RA	0.344**	0.555**	0.324**	0.597**	0.635**	1		
7	T4 IA	0.453**	0.282**	0.451**	0.308**	0.464**	0.323**	1	
8	T4 RA	0.312**	0.508**	0.238**	0.551**	0.303**	0.581**	0.591**	1
	N (Total)	706	706	705	705	734	734	705	705
	Mean	4.273	4.257	4.270	4.127	4.224	4.147	4.241	4.146
	SD	0.960	1.065	0.958	1.127	0.960	1.101	0.954	1.075
	Skew index	−0.578	−0.562	−0.456	−0.568	−0.322	−0.516	−0.507	−0.655
	Kurtosis index	0.906	0.188	0.772	0.270	0.556	0.227	1.180	0.717

**N** varies slightly across waves due to missing data

IA = Individuating Autonomy; RA = Relating Autonomy

**p <0.01

Longitudinally, both IA and RA exhibited moderate stability. IA at T1 correlated with IA at T2 through T4 (*r*s = 0.440–0.489), and RA correlations across adjacent waves (e.g., T2–T3, T3–T4) ranged from 0.581 to 0.597. These patterns indicate partial synchrony within time but underscore the distinct longitudinal stability of each construct, warranting further modeling.

### Measurement Invariance

To ensure valid comparisons over time and across subgroups (gender and educational stage), longitudinal measurement invariance of the autonomy scales was tested sequentially. Configural, metric, and scalar invariance models were examined following established guidelines (Cheung & Rensvold, 2002; Little, 1997; Marsh et al., 2013; Vandenberg & Lance, 2000; Wu et al., 2007; Wu et al., 2010). Model fit was evaluated based on changes in CFI (ΔCFI ≤ 0.02), TLI (ΔTLI ≤ 0.05), and chi-square difference testing (Δ*χ*²), with the scalar invariance model demonstrating acceptable fit across time, gender, and educational stage (see Table 3). These results confirmed the comparability of constructs and justified the use of latent growth modeling and multi-group analysis.

**Table 3:** Testing Longitudinal, Gender and Educational Stage Invariance of Dual Autonomy in Adolescents

	Model fit test statistics and fit indices						Nested Model Comparison Results			
	χ²	*df*	CFI	TLI	RMSEA	SRMR	$\triangle {{\rm{\chi }}}^{2}$	$\triangle$*df*	$\triangle$*CFI*	$\triangle$*TLI*
**Longitudinal Invariance** **Step 1: Configural Invariance**										
1.1	921.36	212	0.934	0.917	0.069	0.051	—	—	—	—
**Step 2: Metric Invariance**										
2.1 ${\Lambda }_{1}={\Lambda }_{2}$	941.96	222	0.933	0.920	0.067	0.053	20.60	10	**0.001**	**−0.003**
2.2${\Lambda }_{1}={\Lambda }_{2}={\Lambda }_{3}$	962.61	232	0.932	0.922	0.066	0.054	20.65	10	**0.001**	**−0.002**
2.3${\Lambda }_{1}={\Lambda }_{2}={\Lambda }_{3}={\Lambda }_{4}$	979.77	242	0.931	0.925	0.065	0.056	**17.16**	10	**0.001**	**−0.003**
**Step 3: Scalar Invariance**										
3.1 ${{\rm{\tau }}}_{1}={{\rm{\tau }}}_{2}$	996.00	252	0.930	0.927	0.064	0.056	—	—	—	—
3.2 ${{\rm{\tau }}}_{1}={{\rm{\tau }}}_{2}={{\rm{\tau }}}_{3}$	1027.31	262	0.929	0.928	0.064	0.056	31.31	10	**0.001**	**−0.001**
3.3 ${{\rm{\tau }}}_{1}={{\rm{\tau }}}_{2}={{\rm{\tau }}}_{3}={{\rm{\tau }}}_{4}$	1055.81	272	0.927	0.929	0.064	0.056	28.5	10	**0.002**	**−0.001**
**Gender Invariance**										
4.1 ${\Lambda }_{{\rm{M}}}\ne {\Lambda }_{{\rm{F}}}\& {{\rm{\tau }}}_{{\rm{M}}}\ne {{\rm{\tau }}}_{{\rm{F}}}$	852.18	106	0.929	0.911	0.070	0.051	—	—	—	—
4.2 ${\Lambda }_{{\rm{M}}}={\Lambda }_{{\rm{F}}}\& {{\rm{\tau }}}_{{\rm{M}}}\ne {{\rm{\tau }}}_{{\rm{F}}}$	892.61	116	0.926	0.916	0.069	0.055	40.43	10	**0.003**	**−0.005**
4.3 ${\Lambda }_{{\rm{M}}}={\Lambda }_{{\rm{F}}}\& {{\rm{\tau }}}_{{\rm{M}}}={{\rm{\tau }}}_{{\rm{F}}}$	951.43	126	0.921	0.917	0.068	0.057	58.82	10	**0.005**	**−0.001**
**Educational Stage Invariance**										
5.1 ${\Lambda }_{{\rm{JH}}}\ne {\Lambda }_{{\rm{S}}{\rm{H}}}\& {{\rm{\tau }}}_{{\rm{JH}}}\ne {{\rm{\tau }}}_{{\rm{SH}}}$	868.05	106	0.929	0.911	0.071	0.051	—	—	—	—
5.2 ${\Lambda }_{{\rm{JH}}}={\Lambda }_{{\rm{SH}}}\& {{\rm{\tau }}}_{{\rm{JH}}}\ne {{\rm{\tau }}}_{{\rm{SH}}}$	899.02	116	0.927	0.917	0.069	0.053	30.97	10	**0.002**	**−0.006**
5.1 ${\Lambda }_{{\rm{JH}}}={\Lambda }_{{\rm{SH}}}\& {{\rm{\tau }}}_{{\rm{JH}}}={{\rm{\tau }}}_{{\rm{SH}}}$	934.80	126	0.924	0.921	0.067	0.053	35.78	10	**0.003**	**−0.004**

Λ represents the factor loading matrix, and τ represents the intercept vector. The subscript numbers indicate the time points of data collection, e.g., Λ_1_ represents the factor loading matrix at the first wave, and so on; 4.1/5.1 denote the freely estimated Λ and τ for male/female and junior high/senior high school groups, respectively; M=male, F=female, JH=junior high school group, SH=senior high/vocational school group; 4.2/5.2 indicate that fixes Λ to be equal across groups while τ remains freely estimated; 4.3/5.3 denote that both Λ and τ are fixed to be equal across groups; Bolded numbers indicate the preferred nested model comparisons (ΔCFI ≤ 0.02 and ΔTLI ≤ 0.05)

### Developmental Trajectories Based on Latent Growth Modeling

To examine the longitudinal development of individuating autonomy (IA) and relating autonomy (RA), five bivariate latent growth models (LGMs) were compared using data from four measurement waves (*N* = 777). IA and RA were assessed using parallel subscales from the same dual autonomy measure. Each observed variable represented a parcel score averaged from six items per wave (e.g., IA1–IA4, RA1–RA4). Latent growth factors for IA and RA were freely estimated to covary, allowing the model to capture potential interrelations in their developmental trajectories. Time scores were fixed at 1.0 for all intercept factors, at 0, 0.5, 1.0, and 1.5 for the linear slope factors, and at 0, 0.25, 1.0, and 2.25 for the RA quadratic slope, capturing the hypothesized curvilinear trajectory of relational autonomy. To account for shared method variance, residual covariances were specified between IA and RA observed variables at each corresponding wave (e.g., IA1 with RA1).

**Fig. 1:**
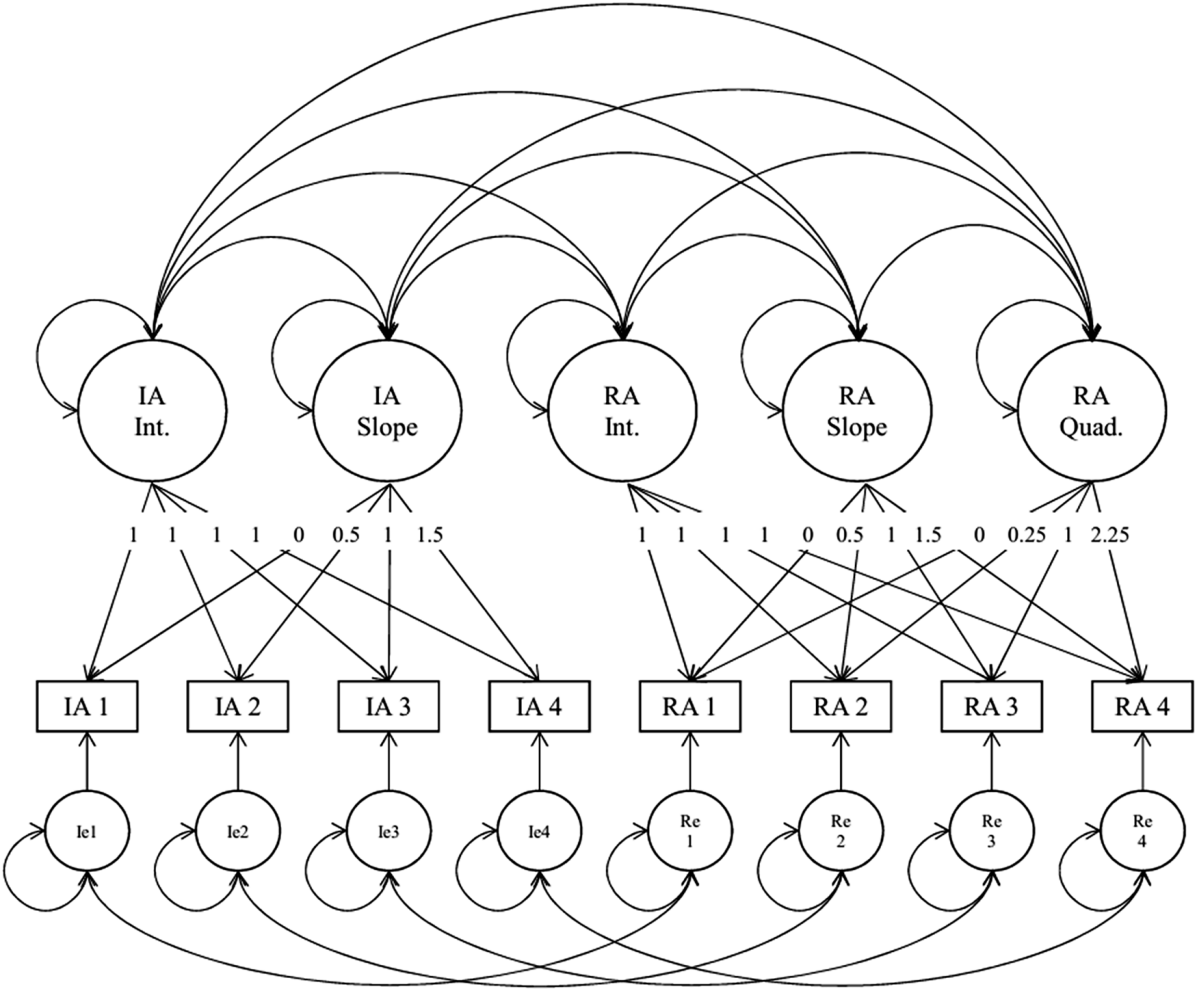
Final Bivariate Latent Growth Model (Model 4) with Linear Growth for Individuating Autonomy (IA) and Quadratic Growth for Relating Autonomy (RA). IA = Individuating Autonomy; RA = Relating Autonomy; Int. = Intercept; Slope = Linear Growth; Quad. = Quadratic Growth; Ie/Re = Residual variances of observed IA/RA variables. IA1–IA4 and RA1–RA4 represent parcel means of six items per wave. Latent growth factors were allowed to covary. Time scores were fixed at 1 for all intercepts, 0, 0.5, 1.0, and 1.5 for slopes, and 0, 0.25, 1.0, and 2.25 for RA quadratic terms. Residual covariances between IA and RA indicators at each wave account for shared method variance

Among these five models, Model 4—which specified a linear growth trajectory for IA and a quadratic growth trajectory for RA—provided the best overall fit (AIC = 13,621.49; CFI = 0.995; RMSEA = 0.029; SRMR = 0.020), striking an optimal balance between parsimony and developmental complexity (see Table 4). The corresponding path diagram is presented in Fig. 1.

**Table 4:** Model fit comparisons across hierarchical latent growth models for individuating and relating autonomy

Models Compared	χ²(A)	df(A)	c(A)	χ²(B)	df(B)	c(B)	Δχ²(S-B)	Δdf	p -value
M1 vs M2	47.054	27	1.3024	37.429	23	1.3174	**9.85**	4	0.043^*^
M2 vs M3	37.429	23	1.3174	35.527	18	1.3423	1.32	5	0.933
M3 vs M4	35.527	18	1.3423	20.083	12	1.3073	**15.18**	6	0.019^*^
M4 vs M5	20.083	12	1.3073	10.418	5	1.1138	10.14	7	0.181

Model comparison proceeds hierarchically: M1 assumes no change over time in either IA or RA (intercept-only model); M2 allows RA to change linearly over time while IA remains constant; M3 specifies linear growth for both IA and RA; M4 models IA as linear and RA as quadratic (nonlinear change); M5 represents the most complex model, assuming quadratic growth for both IA and RA. Δχ² values are computed using the Satorra-Bentler scaled chi-square difference test suitable for MLR estimators. Significant improvement was found between M1 and M2 (*p* < 0.05) and between M3 and M4 (*p* < 0.05), suggesting that adding linear and then quadratic slope terms for RA improved model fit. However, adding a quadratic slope for IA (M4 vs M5) did not significantly enhance model fit (*p* = 0.181)

^*^p < 0.05

In the selected Model 4 (Table 5), the mean intercept for IA was significantly positive (Estimate = 4.254, SE = 0.033, *p* < 0.001), indicating a high initial level. The linear slope was not significant (Estimate = –0.020, SE = 0.025, *p* = 0.416), suggesting overall stability in IA across time. For RA, the mean intercept was also significantly positive (Estimate = 4.223, SE = 0.038, *p* < 0.001). The significant negative linear slope (Estimate = –0.195, SE = 0.075, *p* < 0.01) and marginally significant positive quadratic term (Estimate = 0.091, SE = 0.046, *p* < 0.05) suggest a curvilinear trajectory, characterized by an initial decline followed by partial recovery over time. A strong and significant covariance was found between IA and RA intercepts (r = 0.645, *p* < 0.001), indicating that adolescents with higher levels of IA at baseline also exhibited higher RA. In contrast, covariances between other growth parameters (slopes and quadratics) were not significant, reinforcing the notion that IA and RA followed distinct developmental trajectories. Additionally, residual variances for observed scores across the four time points were all significant (*p*s < 0.001), reflecting individual variability around the estimated growth trajectories. Specifically, the residual variances for IA ranged from 0.460 to 0.491, while those for RA ranged from 0.439 to 0.574. These values suggest that although the growth curves captured general trends, considerable within-timepoint variability remained unexplained, highlighting the presence of individual fluctuations not accounted for by the latent growth factors.

**Table 5:** Unstandardized and Standardized Parameter Estimates for the Final Bivariate Latent Growth Model of Individuating Autonomy (IA) and Relating Autonomy (RA)

	Individuating Autonomy (IA)	Relating Autonomy (RA)
	Estimate (SE)	Estimate (SE)
Means		
Intercept	4.254 (0.033)***	4.223 (0.038)***
Slope	−0.020 (0.025)	−0.0195 (0.075)**
Quadratic	—	0.091 (0.046)*
Variances		
Intercept	0.462 (0.053)***	0.656 (0.112)***
Slope	0.025 (0.040)	0.733 (0.538)
Quadratic	—	0.169 (0.187)
Covariances		
IA Intercept ↔ IA Slope	−0.259 (0.212)	—
RA Intercept ↔ RA Slope	—	−0.032 (0.300)
RA Intercept ↔ RA Quadratic	—	−0.056 (0.333)
RA Slope ↔ RA Quadratic	—	−0.359 (0.288)
IA Intercept ↔ RA Intercept	0.645 (0.068)***	—
IA Intercept ↔ RA Slope	−0.027 (0.161)	—
IA Intercept ↔ RA Quadratic	−0.013 (0.193)	—
IA Slope ↔ RA Intercept	−0.307 (0.284)	—
IA Slope ↔ RA Slope	0.651 (0.619)	—
IA Slope ↔ RA Quadratic	−0.797 (0.997)	—

Means and variances are reported as unstandardized estimates. Covariances among latent growth factors are reported as standardized estimates to aid interpretability. SE = standard error. Dashes (—) indicate parameters not estimated or not applicable

*p < 0.05. **p < 0.01. ***p < 0.001

### Gender-Based Multi-Group LGM

Gender differences in the developmental trajectories of individuating autonomy (IA) and relating autonomy (RA) were examined using a multi-group latent growth model based on the optimal Model 4 (see Table 6). Male adolescents reported higher initial levels of IA (Estimate = 4.43, SE = 0.05, *p* < 0.001) and RA (Estimate = 4.30, SE = 0.06, *p* < 0.001) than female adolescents (IA = 4.07, SE = 0.04, *p* < 0.001; RA = 4.15, SE = 0.05, *p* < 0.001).

**Table 6:** Unstandardized and Standardized Parameter Estimates for the Final Bivariate Latent Growth Model of Individuating Autonomy (IA) and Relating Autonomy (RA), by Gender (Male/Female)

	Individuating Autonomy (IA)	Relating Autonomy (RA)
	Estimate (SE)	Estimate (SE)
Means		
Intercept	4.43 (0.05)***/4.07 (0.04)***	4.30 (0.06)***/4.15 (0.05)***
Slope	−0.04 (0.04)/0.00 (0.03)	−0.13 (0.11)/−0.28 (0.10)**
Quadratic	—/—	0.02 (0.07)/0.18 (0.06)**
Variances		
Intercept	0.42 (0.06)***/0.52 (0.06)***	0.52 (0.16)**/0.85 (0.15)***
Slope	0.001 (†)/0.17 (0.04)***	0.71 (0.88)/1.03 (0.49)*
Quadratic	—/—	0.38 (0.31)/0.001 (†)
Covariances		
IA Intercept ↔ RA Intercept	0.75 (0.13)*** / 0.58 (0.07)***	—/—
IA Slope ↔ RA Intercept	−0.05 (0.06) / −0.24 (0.12)*	—/—
IA Slope ↔ RA Slope	0.08 (0.12) / 0.45 (0.21)*	—/—

Means and variances are unstandardized. Covariances among growth factors are standardized for clarity. Values are reported in the order of male/female

SE = standard error. — = not estimated or not applicable

*p < 0.05. **p < 0.01. **p < 0.001

†Fixed to 0.001 due to negative variance (Heywood case). Model comparison showed no significant difference (ΔCFI < 0.010; SB χ² non-significant), supporting model equivalence. The constrained model also had a lower BIC

In terms of growth, the linear slope for IA was non-significant for both male adolescents (Estimate = –0.04, SE = 0.04, *p* = 0.330) and female adolescents (Estimate = 0.00, SE = 0.03, *p* = 0.980), suggesting general stability in individuating autonomy across genders. For RA, male adolescents showed a non-significant linear trend (Estimate = –0.13, SE = 0.11, *p* = 0.230) and a non-significant quadratic trend (Estimate = 0.02, SE = 0.07, *p* = 0.777). In contrast, female adolescents exhibited a significant negative linear slope (Estimate = –0.28, SE = 0.10, *p* < 0.01) and a significant positive quadratic trend (Estimate = 0.18, SE = 0.06, *p* < 0.01), indicating a curvilinear developmental trajectory—an initial decline followed by gradual recovery.

Regarding variance estimates, intercept variances were significant for both genders and across both constructs (e.g., IA intercept variance: male = 0.42, SE = 0.06, *p* < 0.001; female = 0.52, SE = 0.06, *p* < 0.001), demonstrating heterogeneity in initial levels. Notably, the IA slope variance was non-significant in male adolescents (Estimate = 0.001†) but significant in female adolescents (Estimate = 0.17, SE = 0.04, *p* < 0.001), indicating more variability in IA growth among girls. RA slope variances were high but statistically significant only for female adolescents (Estimate = 1.03, SE = 0.49, *p* < 0.05).

Residual variances for observed IA and RA indicators were largely significant across time points, though smaller in magnitude for female adolescents than male adolescents at some points (e.g., e1_IA: male = 0.61, SE = 0.10, *p* < 0.001; female = 0.23, SE = 0.05, *p* < 0.001), suggesting lower time-specific error among girls.

Covariances between IA and RA intercepts were substantial and significant for both genders (male: *r* = 0.75, *p* < 0.001; female: *r* = 0.58, *p* < 0.001), while other cross-domain covariances were mostly non-significant. One exception was the significant covariance between IA slope and RA intercept for female adolescents (*r* = –0.24, *p* < 0.05), suggesting that girls with lower initial RA tended to show more rapid IA growth. A significant positive association between IA and RA slopes was also found in female adolescents (*r* = 0.45, *p* < 0.05), indicating a co-developmental tendency in this group.

### Educational Stage Differences

The developmental trajectories of individuating autonomy (IA) and relating autonomy (RA) were examined across educational stages using a multi-group latent growth model based on the optimal Model 4 (see Table 7). Junior high school students reported significantly higher initial levels of both Individuating Autonomy (IA: Estimate = 4.35, SE = 0.05, *p* < 0.001) and Relating Autonomy (RA: Estimate = 4.38, SE = 0.05, *p* < 0.001) compared to senior high students (IA = 4.17, SE = 0.05, *p* < 0.001; RA = 4.08, SE = 0.05, *p* < 0.001).

**Table 7:** Unstandardized and Standardized Parameter Estimates for the Final Bivariate Latent Growth Model of Individuating Autonomy (IA) and Relating Autonomy (RA), by Educational Stage (Junior/Senior)

	Individuating Autonomy (IA)	Relating Autonomy (RA)
	Estimate (SE)	Estimate (SE)
Means		
Intercept	4.35 (0.05)*** / 4.17 (0.05)***	4.38 (0.05)*** / 4.08 (0.05)***
Slope	0.01 (0.03) / −0.05 (0.04)	-0.14 (0.11) / -0.25 (0.10)**
Quadratic	— / —	0.06 (0.07) / 0.12 (0.06)*
Variances		
Intercept	0.51 (0.07)*** / 0.39 (0.08)***	0.66 (0.16)*** / 0.59 (0.10)***
Slope	0.03 (0.05) / 0.02 (0.06)	1.42 (0.79) / 0.001 (†)
Quadratic	— / —	0.41 (0.27) / 0.001 (†)
Covariances		
IA Intercept ↔ RA Inter.	0.61 (0.09)*** / 0.65 (0.10)***	— / —

Means and variances are unstandardized. Covariances among growth factors are standardized for clarity. Values are reported in the order of junior/senior

SE = standard error. — = not estimated or not applicable

*p < 0.05. **p < 0.01. **p < 0.001

†Fixed to 0.001 due to negative variance (Heywood case). Model comparison showed no significant difference (ΔCFI < 0.010; SB χ² non-significant), supporting model equivalence. The constrained model also had a lower BIC

In terms of developmental trajectories, IA showed non-significant linear slopes in both junior (Estimate = 0.01, SE = 0.03, *p* = 0.741) and senior students (Estimate = –0.05, SE = 0.04, *p* = 0.235), indicating general stability in individuating autonomy across stages. For RA, junior students exhibited a non-significant linear slope (Estimate = –0.14, SE = 0.11, *p* = 0.189) and a non-significant quadratic trend (Estimate = 0.06, SE = 0.07, *p* = 0.388), suggesting minimal change over time. In contrast, senior students demonstrated a significant negative linear trend (Estimate = –0.25, SE = 0.10, *p* < 0.01) and a significant positive quadratic trend (Estimate = 0.12, SE = 0.06, *p* < 0.05), indicating a U-shaped developmental pattern in RA during later adolescence.

Variance estimates revealed significant heterogeneity in intercepts across both stages and domains (e.g., RA intercept variance for juniors = 0.66, SE = 0.16, *p* < 0.001; seniors = 0.59, SE = 0.10, *p* < 0.001). However, variances for linear and quadratic slopes were small and non-significant, with several parameters fixed (†) due to Heywood cases (e.g., RA slope variance for seniors = 0.001). This suggests limited individual variability in growth trajectories, especially in the senior group.

Residual variances for IA and RA were generally significant across time points. Notably, RA residual variances appeared consistently higher among senior students (e.g., RA e4 = 0.66, SE = 0.14, *p* < 0.001) than juniors (RA e4 = 0.43, SE = 0.18, *p* < 0.05), potentially indicating more time-specific fluctuations in relational experiences among older adolescents.

Covariances among growth parameters were largely non-significant in both groups. Nevertheless, the intercepts of IA and RA remained moderately to strongly correlated (*r* = 0.61 for juniors; *r* = 0.65 for seniors, *p* < 0.001), suggesting a consistent alignment between the two autonomy domains across educational stages.

### Eight-Wave ALGM with Gender as Covariate

Given the established scalar measurement invariance across time and educational stage, and the consistently higher—but declining—autonomy scores among junior high students, the integration of junior and senior high school data into a unified eight-wave structure was both conceptually and empirically warranted.

**Fig. 2:**
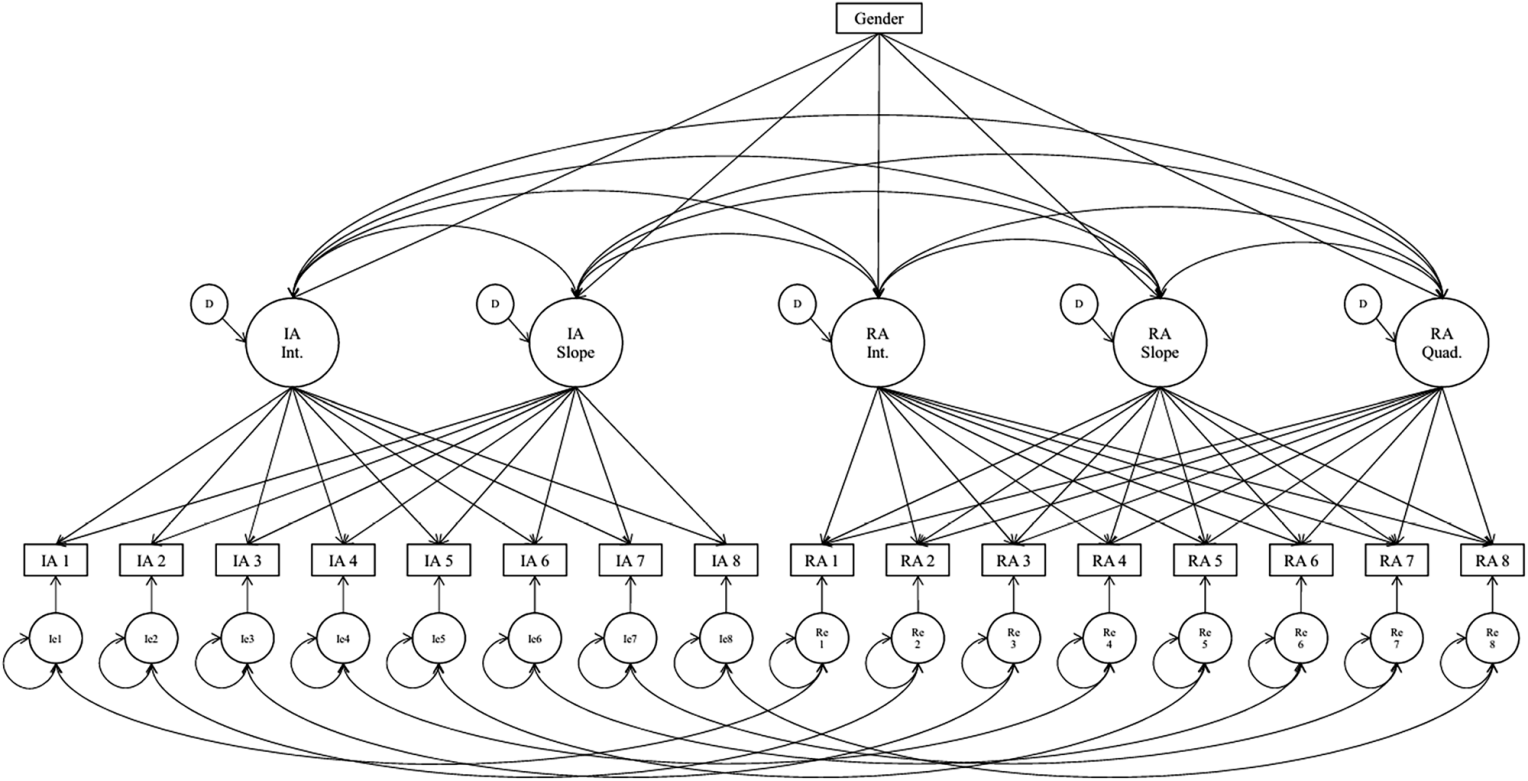
Bayesian Autoregressive Latent Growth Model (ALGM) with Linear Growth for IA and Quadratic Growth for RA Across Eight Waves. IA = Individuating Autonomy; RA = Relating Autonomy; Int. = Intercept; Slope = Linear growth factor; Quad. = Quadratic growth factor; Ie/Re = Residuals of observed IA/RA variables; D = Disturbance terms (latent factor residuals). Time scores: Intercept = 1 (fixed across all waves); Slope = 0, 0.5, 1, 1.5, 3, 3.5, 4, 4.5; Quad. = 0, 0.25, 1, 2.25, 9, 12.25, 16, 20.25. Residual covariances between same-wave IA and RA indicators were specified to account for shared method variance. Gender was included as a time-invariant covariate

The corresponding model is depicted in Fig. 2. The model combines junior high (Waves 1–4) and senior high school data (Waves 5–8) using a planned missing design. IA was modeled with a linear growth factor, whereas RA included both linear and quadratic growth components to capture potential nonlinearities. Time scores for slope factors were set at 0, 0.5, 1, 1.5, 3, 3.5, 4, and 4.5, and those for the RA quadratic component were set as their squared values: 0, 0.25, 1, 2.25, 9, 12.25, 16, and 20.25. Intercept factors for both IA and RA were fixed at 1.0 across all time points. Residual covariances between IA and RA observed variables at the same wave were specified to account for shared method variance. Latent growth factors were allowed to covary, and gender was included as a time-invariant covariate predicting all growth parameters. The model showed adequate fit based on Bayesian posterior predictive checking, with a posterior predictive p-value (PPP) of 0.332 and a 95% confidence interval for the discrepancy between observed and replicated χ² ranging from –36.99 to 60.49, suggesting no substantial discrepancy between observed and model-replicated data (values closer to 0.5 indicate good fit; values near 0 or 1 indicate poor fit) (Asparouhov & Muthén, 2010) .

Autoregressive latent growth model (ALGM) results revealed nuanced developmental trajectories of adolescents’ dual autonomy across eight waves (Table 8). Unstandardized estimates indicated that the initial level of individuating autonomy (IA) was 4.371 (SE = 0.046, *p* < 0.001), with a modest but significant negative linear slope (–0.056, SE = 0.015, *p* < 0.001), suggesting a gradual decline in self-directed autonomy over time. For relating autonomy (RA), the intercept was similarly high at 4.370 (SE = 0.051, *p* < 0.001), and the linear slope was also negative and significant (–0.110, SE = 0.036, *p* < 0.01). In contrast, the quadratic component of RA was non-significant (Estimate = 0.005, SE = 0.007, *p* = 0.230), indicating a lack of strong curvilinear rebound across adolescence.

**Table 8:** Parameter Estimates of the Auto-regressive Latent Growth Model (ALGM) for Individuating Autonomy (IA) and Relating Autonomy (RA) with Gender as a Time-Invariant Predictor

	Individuating Autonomy (IA)	Relating Autonomy (RA)
	Estimate (SE)	Estimate (SE)
Means		
Intercept	4.371 (0.046)***	4.370 (0.051)***
Slope	-0.056 (0.015)***	−0.110 (0.036)**
Quadratic	—	0.005 (0.007)
Variances		
Intercept	0.563 (0.058)***	0.646 (0.080)***
Slope	0.013 (0.008)***	0.140 (0.058)**
Quadratic	—	0.006 (0.002)**
Covariances / Regressions		
IA Intercept ↔ IA Slope	−0.610 (0.084)***	
RA Intercept ↔ RA Slope	—	−0.05(0.21)
RA Intercept ↔ RA Quadratic	—	−0.17(0.23)
RA Slope ↔ RA Quadratic		−0.205 (0.011)***
IA Intercept ↔ RA Intercept	0.618 (0.057)***	—
IA Intercept ↔ RA Slope	−0.068 (0.144)	—
IA Intercept ↔ RA Quadratic	0.002 (0.168)	—
IA Slope ↔ RA Intercept	−0.409 (0.197)*	—
IA Slope ↔ RA Slope	0.394 (0.260)	—
IA Slope ↔ RA Quadratic	−0.232 (0.277)	—
Gender → IA Intercept	−0.253 (0.057)***	—
Gender → IA Slope	0.107 (0.133)	—
Gender → RA Intercept	—	−0.155 (0.063)**
Gender → RA Slope	—	0.102 (0.104)
Gender → RA Quadratic	—	−0.028 (0.098)

Means and variances are unstandardized. Covariances and regression paths involving gender are standardized to facilitate interpretation. SE = standard error. — = not estimated or not applicable

*p < 0.05. **p < 0.01. ***p < 0.001

Variability across individuals was evident in both domains. The variances of intercepts for IA (0.563, SE = 0.058, *p* < 0.001) and RA (0.646, SE = 0.080, *p* < 0.001) were significant, and both linear slopes (IA = 0.013, *p* < 0.001; RA = 0.140, *p* < 0.01) also exhibited significant interindividual differences. The variance in the RA quadratic term was small but statistically significant (0.006, SE = 0.002, *p* < 0.01), suggesting subtle but meaningful variability in the degree of curvature.

Strong within-time residual variances were observed across waves, with generally higher error variances in later waves (e.g., Wave 7: IA = 0.634, RA = 0.549), reflecting increased short-term fluctuation as adolescent’s progress through developmental stages.

Standardized covariances between growth factors provided further insight. IA and RA intercepts were positively correlated (*r* = 0.618, *p* < 0.001), indicating that adolescents who initially viewed themselves as more self-directed also tended to feel more relationally connected. The IA intercept was negatively associated with IA slope (*r* = –0.610, *p* < 0.001), suggesting that adolescents with higher initial levels of IA experienced steeper declines over time. Among RA growth parameters, the correlation between linear and quadratic slopes was significantly negative (*r* = –0.205, *p* < 0.001), indicating that sharper declines in RA were associated with reduced curvature or recovery.

Gender was included as a time-invariant predictor. Boys (coded 1) reported significantly higher initial levels of both IA (*β* = –0.253, SE = 0.057, *p* < 0.001) and RA (*β* = –0.155, SE = 0.063, *p* < 0.01) than girls (coded 2). However, gender did not significantly predict growth rates (slopes or quadratic terms) in either domain, indicating that, although boys and girls differed in baseline levels of autonomy, their developmental trajectories were largely comparable over time.

Overall, the ALGM results depict a landscape in which both forms of autonomy begin high and gradually decline throughout adolescence, with persistent short-term co-fluctuations and shared baseline tendencies, but limited evidence for long-term divergence or curvilinear recovery.

## Discussion

Research on adolescent autonomy continues to face two major challenges: conceptually and empirically understanding how volitional functioning develops within relationally interdependent cultures, and tracing its developmental course across key educational transitions. Although prior studies have demonstrated that autonomy is a universal psychological need (Chirkov, 2011; Ryan & Deci, 2017), few have captured how distinct expressions of volition—those oriented toward personal agency versus relational harmony—change over time within culturally interdependent contexts. Previous efforts have tended to examine autonomy either as a universal construct detached from culture or as a culturally bound construct without addressing its developmental nature (Bao & Lam, 2008; Kagitcibasi, 2005; Soenens et al., 2017), thereby creating a conceptual gap between cultural and developmental approaches. These gaps have limited theoretical integration between developmental and cultural frameworks, leaving unclear how autonomy-as an expression of volitional agency- operates within relationally interdependent contexts. Conceptually, volition refers to the internal psychological process of acting with self-endorsement and a sense of choice, whereas autonomy represents the behavioral manifestation of that volitional process within specific relational and cultural contexts (Deci & Ryan, 2000; Soenens & Vansteenkiste, 2020). To bridge these gaps, this study examined the longitudinal trajectories of individuating and relating autonomy among Taiwanese adolescents, revealing both shared foundations and distinct pathways influenced by cultural and developmental contexts.

The present findings extend the application of Self-Determination Theory (Ryan & Deci, 2017) by illustrating how autonomy operates as volitional action through both individuating and relational processes. Within SDT, autonomy and relatedness are complementary psychological needs that jointly sustain volitional functioning; autonomy is exercised not in isolation but within relationships that enable self-endorsed action. Rather than redefining SDT beyond its Western origins, these findings reaffirm its core premise—that volition flourishes through supportive relationships—while showing how this principle manifests in relationally interdependent cultural contexts. In this sense, the current study contributes nuance rather than contrast: it demonstrates that autonomy’s relational embeddedness, long emphasized within SDT and Western relational theories (e.g., Grotevant & Cooper, 1986; Ryan, 1993), is also developmentally evident in Confucian-heritage contexts where volition and relatedness are co-regulated through family and educational structures.

In this study, individuating autonomy remained stable across adolescence, whereas relating autonomy followed a nonlinear trajectory, particularly among girls and senior high school students. These patterns indicate that autonomy is context-sensitive, shaped by both individual and relational demands. Importantly, autonomy and relatedness were not in conflict: adolescents who displayed higher personal agency also maintained relational attunement, supporting the view that self-directed action can coexist with connectedness when nurtured by supportive relationships. Furthermore, these findings clarify that autonomy differs from independence: adolescents’ autonomous behavior may align with parental expectations when those expectations are internalized rather than externally imposed. This pattern is consistent with cross-cultural findings showing that autonomy-supportive environments promote self-endorsed action even in highly relational settings (Bao & Lam, 2008; Soenens et al., 2017). Together, these results affirm that autonomy, as conceptualized in Self-Determination theory, is relationally enacted and culturally grounded. They align with Western theories (Grotevant & Cooper, 1986; Kagitcibasi, 2005; Ryan, 1993) and East Asian frameworks such as the dual autonomy model (Yeh & Yang, 2006), underscoring that autonomy development is both culturally situated and psychologically universal—an ongoing coordination of self-endorsed action and relational harmony across adolescence.

With respect to the second research question, gender and educational differences in autonomy trajectories highlight how cultural norms and developmental contexts jointly influence the ways adolescents negotiate agency and relational harmony. Both girls and senior high school students reported lower initial levels of relating autonomy and exhibited temporary declines followed by partial recovery, whereas individuating autonomy remained relatively stable across subgroups. These patterns suggest that relational autonomy is particularly sensitive to contextual and gendered expectations in Confucian-heritage societies, where adolescent girls are often socialized to maintain relational harmony and fulfill family obligations alongside academic achievement (Chao, 1994; Way et al., 2014). This interpretation aligns with cross-cultural research indicating that relationally oriented volition is especially responsive to gendered role expectations and changing social demands (Soenens & Vansteenkiste, 2020; Yeh et al., 2016). During the transition to senior high school, the combined pressures of filial duty and academic performance may heighten relational tension, temporarily constraining the volitional aspect of relatedness before adolescents regain balance through adaptive negotiation. By contrast, individuating autonomy which reflects self-endorsed agency. appears less dependent on external conditions. It represents a relatively stable sense of personal efficacy even amid shifting social expectations. Taken together, these findings reveal that relational autonomy, rather than individual agency, is the more adaptable aspect of autonomy in interdependent cultural contexts. More broadly, they underscore that autonomy develops as a flexible process shaped by both relational and developmental contexts.

Finally, to address how autonomy is constructed and differentiated within relationally interdependent contexts (Research Question 3), this study found that the distinct yet coordinated growth of individuating and relating autonomy supports the dual autonomy framework, which conceptualizes autonomy as a balance between self-direction and relationship attunement (Yeh & Yang, 2006). The observed co-fluctuation of these two forms across adolescence suggests that autonomy development in relational cultures is not a linear progression toward independence but a dynamic equilibrium between personal agency and connectedness. This finding aligns with domain-specific and governance-transfer perspectives (Smetana, 2017; Tilton-Weaver & Marshall, 2017), which emphasize that autonomy unfolds unevenly across life domains and is co-regulated through evolving parental and peer roles. Methodologically, combining latent growth modeling with Bayesian autoregressive analysis allowed us to capture both long-term trajectories and short-term fluctuations, revealing that relational autonomy is especially sensitive to social transitions. More broadly, this integrative approach advances developmental science by modeling within-person variability and contextualized adaptation, reflecting a growing emphasis in in autonomy research (Molenaar, 2013; Witherspoon et al., 2023). It also enhances understanding of autonomy as a culturally embedded process, capturing the temporal and relational complexity that shape adolescent development within relational societies.

These findings carry important implications for educational and family practices in relational cultures. Although individuating and relating autonomy in this study was assessed primarily within parent–adolescent relationships, the same volitional principles are relevant to school settings where authority and guidance are similarly negotiated between teachers and students. In school settings, autonomy-supportive climates should recognize that autonomy involves both personal expression and relational attunement. Consistent with autonomy-supportive pedagogy (Reeve, 2016), classrooms that acknowledge students’ perspectives and provide meaningful choices can enhance both agency and relatedness. Teachers can promote autonomy not only by offering choice and rationale but also by encouraging peer dialogue and perspective-taking, helping students pursue personal goals while maintaining social harmony. Such practices are particularly valuable during school transitions, when relational autonomy tends to decline before stabilizing.

In families, parental support for autonomy should combine acknowledgment of adolescents’ perspectives with affirmation of family connectedness. Parents who provide guidance through explanation rather than control, and who balance expectations with trust, can help adolescents sustain both self-endorsed agency and relational responsibility. These culturally responsive practices align with the dual autonomy framework, suggesting that psychological well-being arises when youth experience volition within enduring relationships rather than apart from them.

Several limitations should be considered when interpreting these findings. First, although the cohort-sequential design extended developmental coverage across adolescence, it did not allow full within-person tracking across all eight waves, limiting insight into short-term individual fluctuations between individuating and relating autonomy. Second, reliance on self-report data may have inflated associations due to shared method variance, underscoring the need for multi-informant or behavioral assessments of volitional functioning. Third, the present study focused on developmental trajectories rather than antecedents or outcomes; future research should therefore investigate how culturally embedded interpersonal factors, such as parenting, peer relations, and school environments shape these dual pathways of volition. Prior longitudinal findings from East Asian contexts suggest that parental control strategies may influences on individuating and relating autonomy in divergent ways: behavioral guidance from fathers often predicts subsequent gains in adolescents’ self-endorsed agency, whereas psychological control from mothers tends to undermine relational autonomy over time (Kuo, 2022). Moreover, as educational transitions into new school contexts, peer and teacher autonomy support may further affect how they balance agency with relational connectedness (Liu, 2013). Future research should integrate these family and educational influences within cross-cultural and longitudinal designs to clarify whether the balance between agency and relatedness reflects universal developmental processes or culturally specific adaptations. Addressing these issues will further refine a culturally inclusive framework for understanding autonomy development.

## Conclusion

Understanding how adolescents develop autonomy within relationally interdependent contexts remains a central challenge for developmental science. Existing models have yet to capture how volitional functioning unfolds when it must coexist with relational responsibilities. Using longitudinal and Bayesian models with Taiwanese youth, the present findings revealed that individuating autonomy remained stable, whereas relating autonomy fluctuated across adolescence—particularly during the transition to senior high school and among girls. These differentiated trajectories indicate that autonomy in interdependent cultures is not a linear progression toward independence but a dynamic balance between self-endorsed action and relational attunement. The findings bridge Self-Determination Theory with relationally embedded conceptions of autonomy, clarifying how self-endorsed action and connectedness jointly support adolescent adaptation. Ultimately, this research offers a culturally inclusive framework for understanding autonomy—one that locates freedom not in separation from others, but in acting authentically within enduring relationships.

## Declarations

### Data Sharing Declaration

The datasets generated and/or analyzed during the current study are not publicly available due to participant confidentiality and restrictions approved by the institutional review board, but are available from the corresponding author on reasonable request.
